# Microleakage in indirect onlay restorations cemented with three different types of adhesives: An *in vitro* study

**DOI:** 10.4317/jced.60725

**Published:** 2023-08-01

**Authors:** Isabel Sinche-Ccahuana, Marysela Ladera-Castañeda, Elizabeth Paucar-Rodríguez, Ana Aliaga-Mariñas, Giannina Dapello-Zevallos, Luis Cervantes-Ganoza, César Cayo-Rojas

**Affiliations:** 1 Universidad Nacional Federico Villarreal, Faculty of Dentistry and Postgraduate School, “Salud Pública – Salud Integral”, Lima, Peru; 2 Universidad Privada San Juan Bautista, School of Stomatology, Lima, Peru; 3 Universidad Inca Garcilaso de la Vega, Faculty of Stomatology, Lima, Peru

## Abstract

**Background:**

To evaluate the *in vitro* degree of marginal microleakage in indirect Class II onlay restorations cemented with dual self-adhesive, universal adhesive and dual adhesive.

**Material and Methods:**

In the present in vitro experimental study, a total of 54 human premolar teeth were prepared and divided into three equal groups (n = 18) for placement of onlay-type restorations cemented with A: Allcem™ dual-cure adhesive cement), B: RelyX™U200 dual-cure self-adhesive cement and C: RelyX™ Ultimate universal adhesive cement. All restorations were subjected to 10,000 thermocycles between 5°C and 55°C and immersed in a 1M silver nitrate solution for 6 hours. The crowns were then sectioned mesiodistally and observed under a stereo microscope to determine the degree of marginal microleakage in the cervical area.

**Results:**

The onlay restorations cemented with RelyX Ultimate did not present microleakage in the majority of cases (77.8%). Restorations cemented with RelyX U200 showed predominantly microleakage up to the pulp floor in 83.3% of the total, being this significantly higher microleakage than in restorations cemented with RelyX Ultimate and Allcem Dual (*p*<0.001 and *p*<0.001 respectively). There was no significant difference in microleakage between the last two mentioned cements (*p*=0.255)

**Conclusions:**

Allcem dual adhesive cement and RelyX Ultimate universal adhesive showed significantly less microleakage than RelyX U200 dual-curing self-adhesive cement at the cervical level, with predominantly no microleakage and microleakage down to the enamel, respectively. The use of RelyX Ultimate cement in indirect restorations is recommended as it showed better marginal adaptation.

** Key words:**Microleakage, human teeth, adhesion, adhesive cement, thermal cycling, onlay restoration.

## Introduction

Indirect restorations are a conservative treatment option for posterior teeth with extensive caries or fractures that do not require a crown (1). There are several criteria and techniques to improve marginal adaptation in indirect restorations in order to protect the dentin-pulp system and the restoration (2). For an adequate marginal adaptation, the formation of microgaps at the enamel-dentin interface should be avoided as much as possible in order to attenuate the risk of bacterial infiltration or food debris (3).

Microleakage at the tooth-restoration interface is considered the main cause of clinical failure of a restoration (4). Therefore, it is associated with clinical manifestations involving postoperative hypersensitivity, recurrent caries, marginal pigmentation of the restoration and even pulp pathology (5).

Currently, dual etch cements are dual-cure resin materials with photopolymerized and chemically cured activation (6). This dual adhesive system is composed of methacrylate monomers such as BisGMA (bisphenol glycidyl methacrylate), BisEMA (bisphenol-A ethoxylated dimethacrylate), TEGDMA (triethylene glycol dimethacrylate), camphorquinone, barium-aluminum-silicate glass microparticles, silica dioxide nanoparticles, inorganic pigments and also benzoyl peroxide (7). Compared to the dual self-adhesive system, they are composed of bifunctional methacrylates that allow them not to require total etching and facilitate adequate bonding performance (8).

Universal cements bond to indirect restorations in a self-curing and light-curing manner, making a dual-curing compatible with total-etch and self-etch universal adhesives (5). Resinous universal cements are composed of monomers such as Bis-GMA, low molecular weight TEGDMA, and hydrophilic functional groups such as HEMA (2-hydroxyethyl methacrylate) and 4-META (4-methacryloyloxyethyl trimellitate) that participate in dentin bonding (9). In addition, they may contain the monomer MDP (10-methacryloyloxyloxyalkyl dihydrogen phosphate) that also promotes adhesion and chemical bonding with the calcium present in the dentin tissue hydroxyapatite, giving greater stability than other monomers present in different cementing agents (10). The incorporation of 10-MDP establishes a chemical integration to the dentin achieving longer duration and less postoperative sensitivity (11).

Inlay-type restorations are characterized by having a large number of angles to prepare, which usually results in an internal adjustment for precise seating (12). Larger occlusal width preparations such as onlay restorations may have a better fit than the smaller preparations used in inlay-type restorations. This is because onlay restorations cover more tooth surface area and may provide greater stability, durability, and support (12). It has also been documented that onlay-type indirect partial restorations are more durable when posterior teeth are extensively restored due to loss or defect of their tooth tissue. Based on this concept, those cusps not supported by dentin that are thin or weakened should be reduced in order to increase the durability of the restoration and prevent tooth fracture. In this way, biomechanical principles are respected to preserve as much healthy tooth tissue as possible (13). It should be considered that the long-term clinical success of indirect restorations is largely determined by the bonding efficacy of the cementing agent (14).

Therefore, the purpose of the present study was to evaluate the degree of microleakage in indirect onlay restorations cemented with dual self-adhesive, universal adhesive and dual adhesive. It was considered as null hypothesis that there are no significant differences in the degree of microleakage when comparing the three adhesive cements mentioned above.

## Material and Methods

-Study design

This experimental *in vitro* and analytical study was performed at the Universidad Nacional Federico Villarreal (UNFV) in Lima - Peru between July and September 2022, with approval letter No. 0117-2022-DAV-FO-UNFV. This study considered the CRIS Guidelines (Checklist for Reporting *In-vitro* Studies).

-Sample calculation and selection

**Figure 1:**
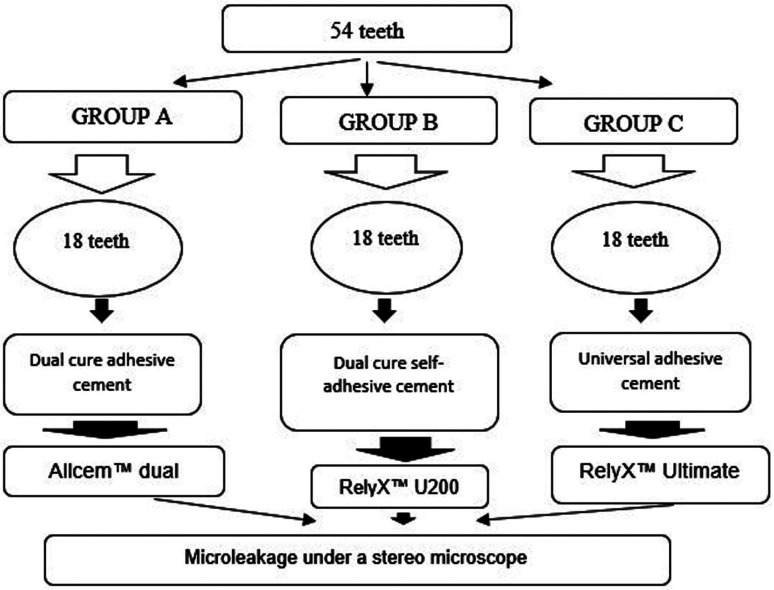
Random distribution of groups according to sample size.

Fifty-four healthy human premolars extracted for orthodontic reasons in the last 3 months prior to the experiment were selected. The teeth were voluntarily donated under informed consent for research purposes respecting the Declaration of Helsinki. Class II onlay cavities were prepared. The sample size per group was 18 teeth (n = 18) and was calculated with G*Power 3.1.9.7 statistical software using an independent proportion comparison formula based on a pilot study where P1 = 0.125 and P2 = 0.500 values were obtained with a significance (α) = 0.05 and statistical power (1 - β) = 0.80. The teeth were randomly distributed into three groups (A, B and C) as follows (Fig. 1):

• Group A: Allcem dual-cured adhesive cement (FGM, São Paulo, Brazil).

• Group B: RelyX™ U200 Dual Cure Self-Adhesive Cement (3M ESPE, St. Paul, MN USA).

• Group C: Universal adhesive cement RelyX™ Ultimate (3M ESPE, St. Paul, MN, USA).

-Sample preparation

**Figure 2:**
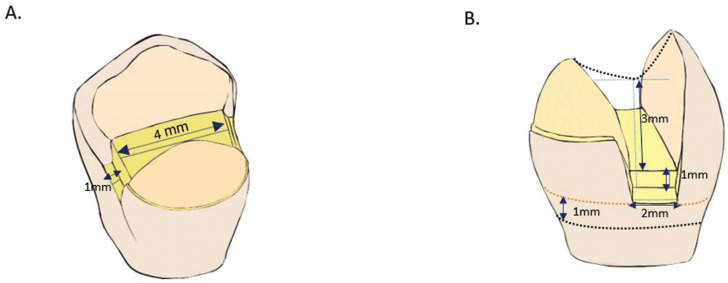
Preparation of Class II cavities. A: Longitudinally sectioned crown (mesio-distal). B: Proximal view.

The teeth were cleaned with Gracey Curettes No 13-14 (Hu-Friedy®, Dentaltix, USA), water and prophylactic brush, removing all organic debris for adhesion. They were disinfected by immersing them in 1% chloramine-T trihydrate solution (Scharlab, ExpertQ®, Barcelona, Spain) for one week. They were then preserved under refrigeration at 4°C with distilled water that was changed every 7 days to minimize deterioration of the samples according to the international standard PD ISO/TS 11405:2015 (15,16). All cavity preparations were performed by the same operator with rounded tip diamond cylindrical bur 446KR.011 (Jota 1925, Ituren, Switzerland, Switzerland) and the dimensions were verified with periodontal probe (North Caroline, Hu-Friedy®, USA) by two investigators as seen in Figure 2. A different bur was used for cavity preparation of each tooth using a high-speed handpiece (NSK PanaMax®, Tokyo, Japan) and abundant irrigation (15).

-Cavity conditioning and cementation of restorations

The cavity surface was prepared using a prophylactic brush with a pumice stone, washed with abundant water in depth for 5 seconds and partially dried with pieces of sterile gauze (ALKHOFAR®, Lima, Peru). The internal surface of all ceromer restorations was sandblasted with 50-micron aluminum oxide at 60/80 PSI (pounds-force per square inch) pressure.

For group A, the surfaces of the cavities were conditioned with 35% orthophosphoric etching acid (Etching Gel Densell®, Buenos Aires, Argentina) for 15 seconds. They were then washed with abundant water for 30 seconds and dried with pieces of sterile gauze. Each restoration was etched with 35% orthophosphoric acid for 1 minute, washed with plenty of water for 30 seconds and finally dried with air pressure for 10 seconds (17). After etching, silane (Silane-X, PresvestDentPro®, India) was applied to the inner surface of the restorations with the help of a micro applicator (DISPOCARE®, Shanghai, China). The solvent was then evaporated with gentle air pressure for 5 seconds. Inside the cavity, Ambar Universal adhesive was applied with a micro applicator and light cured for 20 seconds. The indirect onlay restorations were then cemented with dual-cure adhesive (Allcem dual) and the excess was removed with a micro applicator. Then, glycerin (Alkofarma, Lima, Peru) was placed on the edge of the tooth-restoration interface and light cured using a polywave LED lamp (Woodpecker, Guangxi, China) at a light intensity of 1000 mW/cm2 for 20 sec on each surface (17). Finally, it was polished with flexible spiral brush (QSWTITAN, Shanghai, China) and polishing brush (BADER®, Pontevedra, Spain) using Diamond Polish Mint paste (Ultradent™, South Jordan, USA).

For group B, the teeth and restorations were not acid-etched. Only the tooth surface was prepared with pumice paste because the cement used is a self-adhesive. Then silane was applied on the surface of the restorations with gentle air pressure for 5 seconds to evaporate the solvent. The indirect onlay restorations were cemented with RelyX U200 dual cure self-adhesive, which was mixed manually on a waxed paper block (3M ESPE, St. Paul, MN USA) with a plastic spatula (Dentaltix, Madrid, Spain). The cement was distributed on the surface of the restorations by manually pressing it on the tooth preparation, keeping it firmly in place and then removing the excess with a micro applicator. Next, glycerin was placed on the edge of the interface and light cured for 20 seconds on each surface using a polywave LED lamp at a light intensity of 1000 mW/cm2 (17). Subsequently, polishing was carried out with Diamond Polish Mint paste.

For group C, the surfaces of the cavities were conditioned with 35% orthophosphoric etching acid for 15 seconds. They were then washed with abundant water for 30 seconds and dried with pieces of sterile gauze. Each restoration was etched with 35% orthophosphoric acid for 1 minute, washed with plenty of water for 30 seconds and finally dried with air pressure for 10 seconds (17). After etching, silane was applied on the surface of the restorations and the solvent was evaporated with gentle air pressure for 5 seconds. Then the Singlebond® Universal adhesive was applied with the help of a micro-applicator using gentle air pressure for 5 seconds to evaporate the excess solvent without light curing. The indirect onlay restorations were then cemented with RelyX™ Ultimate universal adhesive. This cement was mixed manually on a block of waxed paper with a plastic spatula. The cement was distributed on the surface of the restorations and manually pressed to seat the restoration to the tooth, removing the excess with the help of a micro applicator. Glycerin was then placed on the edge of the cementation interface and finally, each surface was light cured for 20 seconds using a polywave LED lamp at a light intensity of 1000 mW/cm2 (17). Finally, it was polished with Diamond Polish Mint paste.

-Thermocycling, preparation and immersion of teeth in dye

The restored teeth were subjected to 10,000 thermal cycles in water between 5°C and 55°C. The exposure to each bath was 30 seconds and the transfer time between baths was 10 seconds (18). Nail varnish was applied to all root surfaces and then the apices were covered with self-curing acrylic (Vitacryl, Lab Xpress, Lima, Peru) to avoid dye seepage through the apical foramen (18). The samples were immersed in 1M silver nitrate solution contained in amber glass vials wrapped with aluminum foil for 24 hours without exposure to light and at room temperature. Then these samples were washed with plenty of water for 5 minutes and then immersed in a photoreflective solution under fluorescent light for 8 hours (18). Finally, each sample was rinsed and checked to ensure that the dye had not leached through the apex.

-Sectioning of samples for observation under the stereo microscope

The roots of the restored teeth were cut 3 mm below the cementum-enamel junction (18). In the coronal portion, a longitudinal sectioning was performed in mesio-distal direction to obtain two parts according to the international standard PD ISO/TS 11405:2015 (15,16). For sectioning, 0.20 mm thick bioactive diamond cutting discs were used, one for each tooth, with a low-speed micromotor (Marathon SDE-H37L1, Saeyang, Korea) and abundant irrigation. The sectioned surfaces were then polished with silicon carbide papers using plenty of water for 2 min and dried for observation under a binocular stereo microscope (Leica EZ4, Wetzlar, Germany) at 20x magnification. Stereo microscope readings were performed by a single investigator. To reduce microfiltration reading biases, intraexaminer (k = 0.88; CI: 0.67 - 1.00) and interexaminer (k = 0.76; CI: 0.43 - 1.00) calibration was performed using Cohen’s Kappa index, obtaining good agreement. In addition, the double-blind method was applied since both the statistician and the researcher who performed the stereo microscopic readings were unaware of the group assignment. To measure the degree of microleakage, the scoring system given by the Organization for Standardization PD ISO/TS 11405:2015 international standard was used (15), (Table 1).

**Table 1:**
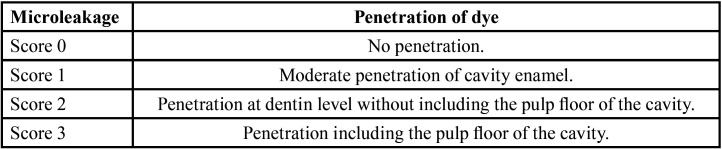
Degree of marginal microleakage according to the penetration of dye.

-Statistical analysis

The data were imported by SPSS version 28.0 statistical software from a Microsoft Excel 2019 spreadsheet. Absolute and relative frequency Tables were used for descriptive analysis. For measures of central tendency and dispersion, the median and interquartile range were used, respectively. For the inferential analysis, the Kruskal Wallis test with the Bonferroni adjustment test was used to compare the degree of microleakage in the three types of adhesive cements by cervical area, considering a significance level of *p*<0.05.

## Results

**Figure 3:**
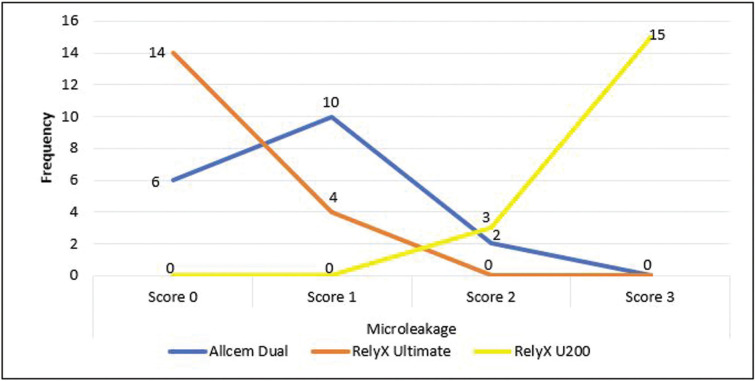
Sample size distribution according to the degree of microleakage in each group of adhesive cement.

Of the 18 samples in each group of adhesive cement, 55.6% of the onlay restorations cemented with Allcem Dual presented grade 1 microleakage. The onlay restorations cemented with RelyX Ultimate did not present microleakage (Grade 0) in the majority of cases (77.8%). Finally, the onlay restorations cemented with RelyX U200 presented predominantly grade 3 microleakage in 83.3% of the total, being this self-adhesive cement the group that presented the most severe microleakage values, (Table 2, Fig. 3).

**Table 2:**
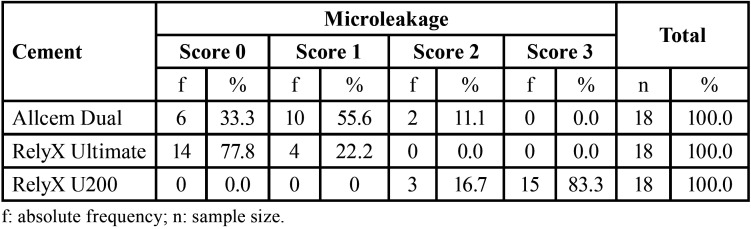
Microleakage degree of adhesive cements.

When onlay restorations cemented with three different types of cements were compared, it could be seen that those cemented with RelyX U200 self-adhesive dual adhesive presented significantly greater microleakage than those cemented with RelyX Ultimate and Allcem Dual (*p*<0.001 and *p*<0.001 respectively). In addition, no significant differences in the degrees of microleakage were evident between onlay restorations cemented with Allcem Dual and RelyX Ultimate universal adhesive (*p* = 0.255), (Table 3).

**Table 3:**
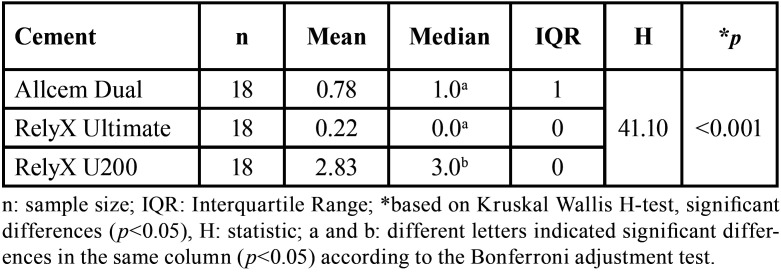
Comparison of microleakage degrees among adhesive cements.

## Discussion

The present study evaluated the degree of marginal microleakage at the cervical level in indirect Class II onlay cement-retained restorations with dual-adhesive, universal adhesive and dual self-adhesive cements. As a result, significantly more microleakage was observed in the cervical area with the dual self-adhesive cement compared to the dual adhesive and universal adhesive cements, rejecting the null hypothesis.

The use of RelyX Ultimate universal adhesive cement showed a lower degree of microleakage in the cervical area compared to RelyX U200 dual self-adhesive cement. This is probably due to the application of the Single BondTM Universal adhesive system which in its chemical composition includes water, HEMA, Vitrebond™ Copolymer, MDP (methacryloyloxyalkyl phosphate) monomer and silane that provide maximum adhesion at the enamel, dentin and restoration level as well as better marginal adaptation at the interface (17). RelyX U200 cement is self-adhesive because it contains methacrylate monomers modified with multifunctional phosphoric acid, which gives it this characteristic, thus replacing acid conditioning. The latter could favor the failure to achieve optimal adhesion and allow microleakage because self-adhesive cements have lower acidity in their composition than orthophosphoric acid and do not achieve such deep conditioning in either enamel or dentin (13,16,19). Another possible explanation for the good seal of RelyX Ultimate could be that the phosphate monomer (MDP) present in the Single-Bond Universal adhesive increases the resistance to biodegradation of the adhesive interface formed from some calcium nanolayers bound to the MDP. In this way the collagen fibers are protected from the hydrolysis process as these nano-layers have been reported to provide high bonding stability and physical strength (9).

Very little microleakage was observed with no statistically significant differences between Allcem Dual cement and RelyX Ultimate cement, probably because these universal adhesive cements contain 10-MDP (10-methacryloyloxydecyl dihydrogen phosphate) which favors the formation of a strong chemical bond between the dental substrate and the restorative material, which is in agreement with several studies (17,19,20).

According to Ilie *et al*. (21), RelyX U200 self-adhesive cement contains acidic and hydrophilic monomers that play an important role in controlling the chemical polymerization reaction. However, the limited information available on the initiator systems hinders a clear interpretation of the behavioral pattern of this material after polymerization (21,22). Also, other studies have reported that acidic monomers present in self-adhesive cements have had a negative effect on the degree of microleakage since they apparently interfere chemically with the amine initiator, which may affect the rate and degree of polymerization (23).

As a strength in the design of the present study, it can be mentioned that 10,000 thermal cycles were performed, since it has been reported that this amount is equivalent to one year of clinical aging in the oral cavity (18,24,25). In addition, the present study used 1M silver nitrate as the dye solution (24,26) because it is one of the most commonly used dyes in micro- and nanofiltration studies. This is due to the fact that silver ions present good diffusion capacity through the tooth-resin interface and absorb light reducing the diamine silver ions with 0.059 nm diameter to metallic silver grains thus making them easier to observe under the stereo microscope (18,27). Another advantage of metallic silver grains is that they are not water-soluble, which does not allow their removal when abundant water washing is used, thus reducing observation biases. Likewise, the present study prepared the proximal box 1 mm above the enamel-cement junction because it has been reported that cervical microleakage at 1 mm below the enamel-cement junction is significantly higher than at 1 mm above. For this reason, adhesion to enamel with acid etching is better than adhesion to cement because enamel has a higher inorganic composition (95%) and less moisture (18,27).

In the present study, glycerin was applied at the edge of the tooth-restoration interface with the aim of preventing the formation of the oxygen-inhibited layer at the interface surface (28). Bergman *et al*. (29) confirmed through *in vitro* experiments that the resin cement surface at the edge of clinical crowns forms a soft and sticky oxygen inhibitory layer, causing poor edge quality of such restorations after cleaning. Therefore, De Munck *et al*. (30) recommend the use of glycerin in the restoration before light curing so that there is no contact of ambient oxygen with the resin cement. In addition, the subsequent removal of glycerin is easy since it is soluble in water.

The importance of the present study lies in comparing three types of adhesive cement and identifying which one obtains better marginal adaptation. It was observed that RelyX Ultimate cement improved the marginal adaptation of the tooth-restoration interface. The application of RelyX Ultimate as a dual-curing cement combined with Single Bond Universal adhesive facilitated better adaptation by creating an intimate bond between the cement and the surrounding dentin, which could contribute to the longevity of the restoration and reduce its clinical failure (4) that often involves postoperative hypersensitivity, recurrent caries, marginal pigmentation of the restoration and even pulpal pathology (5).

The present study was limited to evaluating microleakage under the stereo microscope without the use of scanning electron microscopy (SEM) with energy dispersive X-ray spectroscopy (EDS) since the purpose was not to quantify the amount of silver ions at the tooth-resin interface but to determine the degree of deepening of silver nitrate through the interface. To meet this objective, it was decided to evaluate the marginal microleakage of the restorations under a stereo microscope since this methodology is supported by numerous studies (18,24,26,27). Another limitation in the present study was its performance on *in vitro* teeth with artificial aging, so the results obtained should be taken with caution due to the existence of studies indicating that *in vitro* results are not always extrapolable to clinical results. However, due to the limited clinical evidence comparing the three adhesive cements used in the present study, it is necessary to recommend randomized controlled clinical trials that analyze the microleakage of indirect restorations using dual adhesive, universal adhesive and dual self-adhesive cements of different commercial brands. In addition, it would be advisable to evaluate the microleakage of these adhesive cements with and without the application of glycerin at the edge of the interface surface to verify if the changes are statistically significant.

## Conclusions

Considering the limitations of the present *in vitro* study, it can be concluded that restorations cemented with RelyX™ U200 dual self-adhesive showed a significant increase of microleakage in the cervical area compared to Allcem dual adhesive and RelyX™ Ultimate universal adhesive cements which showed predominantly no microleakage and microleakage down to the enamel respectively. It is advisable to use RelyX Ultimate cement in indirect restorations in order to obtain a better marginal seal.

## References

[1] Tommaso G, Rizcalla N, Krejci I, Dietschi D (2015). Evidence-based concepts and procedures for bonded inlays and onlays. Part II. Guidelines for cavity preparation and restoration fabrication. The International Journal of Esthetic Dentistry.

[2] Salguero J, Altamirano N (2020). Prevalence of dental hypersensitivity after applying immediate dentinary sealing in the fixed partial prosthesis. Journal of American Health.

[3] Carvalho MA, Lazari-Carvalho PC, Polonial IF, Souza JB, Magne P (2021). Significance of immediate dentin sealing and flowable resin coating reinforcement for unfilled/ lightly filled adhesive systems. Journal of Esthetic and Restorative Dentistry.

[4] Samartzi TK, Papalexopoulos D, Sarafianou A, Kourtis S (2020). Immediate Dentin Sealing: A Literature Review. Clin Cosmet Investig Dent.

[5] Gupta A, Tavane P, Gupta PK, Tejolatha B, Lakhani AA, Tiwari R (2017). Evaluation of microleakage with total etch, self-etch and universal adhesive systems in Class V restorations: An in vitro study. J Clin Diagn Res.

[6] Manso AP, Carvalho RM (2017). Dental Cements for Luting and Bonding Restorations Self-Adhesive Resin Cements. Dental Clinics of North America.

[7] Kurt A, Altintas SH, Kiziltas MV, Tekkeli SE, Guler EM Kocyigit A, Usumez A (2018). Evaluation of residual monomer release and toxicity of self-adhesive resin cements. Dental Materials Journal.

[8] Miotti LL, Follak AC, Montagner AF, Pozzobon RT, Da Silveira BL, Susin AH (2020). Is Conventional Resin Cement Adhesive Performance to Dentin Better Than Self-adhesive? A Systematic Review and Meta Analysis of Laboratory Studies. Operative Dentistry.

[9] Moncada G, García FR, de Oliveira OB, Fernández E, Martín J, Vildósola P (2014). Rol del 10-metacriloxidecilfosfato dihidrogenado en el cambio de paradigma de los sistemas adhesivos integrados en la dentina. Revista Clínica de Periodoncia, Implantología y Rehabilitación Oral.

[10] Delgado AHS, Owji N, Ashley P, Young AM (2021). Varying 10-methacryloyloxydecyl dihydrogen phosphate (10-MDP) level improves polymerization kinetics and flexural strength in self-adhesive, remineralising composites. Dental Materials.

[11] Yuan X, Wang Q, Han F, Chen C, Xie H (2021). Chemical interaction between 10-methacryloyloxydecyl dihydrogen phosphate and methacryloxypropyltrimethoxy silane in one-bottle dental primer and its effect on dentine bonding. Journal of the Mechanical Behavior of Biomedical Materials.

[12] Fonseca RB, Correr-Sobrinho L, Fernandes-Neto AJ, Quaglialatto PS, Soares CJ (2008). The influence of the cavity preparation design on marginal accuracy of laboratory-processed resin composite restorations. Clin Oral Invest.

[13] Belleflamme MM, Geerts SO, Louwette MM, Grenade CF, Vanheusden AJ, Mainjot AK (2017). No post-no core approach to restore severly damaged poste- rior teeth: An up to 10-year retrospective study of documented endocrown cases. J Dent.

[14] Manso AP, Carvalho RM (2017). Dental Cements for Luting and Bonding Restorations: Self-Adhesive Resin Cements. Dent Clin North Am.

[15] (2015). Dentistry-Testing of adhesion to tooth structure. https://cdn.standards.iteh.ai/samples/62898/c8afc682cda547d797cf339c726837ce/ISO-TS-11405-2015.pdf.

[16] Cayo CF, Carrillo AAC (2020). Marginal sealing applying sodium hypochlorite versus phosphoric acid as dental conditioner. Rev Cubana Estomatol.

[17] Risco TJG, Álvarez EJÁ (2019). Marginal microleakage in table top ceromer inlays cemented with resinous cements: self-etching, universal and thermoplasticized resin. Revista Odontología.

[18] Cayo-Rojas C, Hernández-Caba K, Aliaga-Mariñas A, Ladera-Castañeda M, Cervantes-Ganoza L (2021). Microleakage in class II restorations of two bulk fill resin composites and a conventional nanohybrid resin composite: an in vitro study at 10,000 thermocycles. BMC Oral Health.

[19] Moreno LR, Saavedra ADM, Limón BR (2020). Comparison of bond strength to dentin of self-etching cements vs total Etching. Revista ADM.

[20] Luz ME, Tichy A, Hosaka K, Ikeda M, Nakajima M, Tagami J (2021). The effect of curing mode of dual-cure resin cements on bonding performance of universal adhesives to enamel, dentin and various restorative materials. Dental Materials Journal.

[21] Ilie N, Simon A (2012). Effect of curing mode on the micro-mechanical properties of dual-cured self-adhesive resin cements. Clin Oral Investig.

[22] Urcuyo AMS, Escobar GDM, Pozos GA, Flores AJC, Romo RGF, Ortiz MM (2020). Evaluation of the Bond Strength and Marginal Seal of Indirect Restorations of Composites Bonded with Preheating Resin. European Journal of Dentistry.

[23] Frassetto A, Navarra CO, Marchesi G, Turco G, Di Lenarda R, Breschi L (2012). Polymerization kinetics and development of shrinkage stresses in self-adhesive resinous cements. Dent Mater.

[24] Mosharrafian S, Heidari A, Rahbar P (2017). Microleakage of Two Bulk Fill and One Conventional Composite in Class Two Restorations of Primary Posterior Teeth. J Dent.

[25] Alcántara-Obispo E, Santander-Rengifo F, Ladera-Castañeda M, López-Gurreonero C, Castro Pérez-Vargas A, Cornejo-Pinto A (2022). Adhesive Strength in Dentin Conditioned with 18% Ethylenediaminetetraacetic Acid versus 35% Phosphoric Acid: In Vitro Study with 1-Year Artificial Aging. Polymers.

[26] Miletic V, Peric D, Milosevic M, Manojlovic D, Mitrovic N (2016). Local deformation fields and marginal integrity of sculptable bulk-fill, low-shrinkage and conventional composites. Dent Mater.

[27] López-Torres J, Hernández-Caba K, Cervantes-Ganoza L, Ladera-Castañeda M, Martínez-Campos R, Solís-Dante F (2023). Microleakage of Class II Bulk-Fill Resin Composite Restorations Cured with Light-Emitting Diode versus Quartz Tungsten-Halogen Light: An In Vitro Study in Human Teeth. Biomedicines.

[28] Wen-xin CH, Xu-dong B, Lin Y (2020). Curing method affecting the formation of oxygen inhibition layer on the surface of resin cement. Journal of Peking University.

[29] Bergman P, Noack M, Roulet JF (1991). Marginal adaptation with glass-ceramic inlays adhesively luted with glycerine gel. Quintessence International.

[30] De Munck J, Van Landuyt K, Peumans M, Poitevin A, Lambrechts P, Braem M (2005). A critical review of the durability of adhesion to tooth tissue: methods and results. J Dent Res.

